# Analysis of Immune Response Markers in Jorge Lobo's Disease Lesions Suggests the Occurrence of Mixed T Helper Responses with the Dominance of Regulatory T Cell Activity

**DOI:** 10.1371/journal.pone.0145814

**Published:** 2015-12-23

**Authors:** Michelle de C. S. Azevedo, Patricia S. Rosa, Cleverson T. Soares, Luciana R. V. Fachin, Ida Maria F. D. Baptista, William J. Woods, Gustavo P. Garlet, Ana Paula F. Trombone, Andrea de F. F. Belone

**Affiliations:** 1 Departamento de Doenças Tropicais, Faculdade de Medicina de Botucatu, Universidade Estadual Paulista, Botucatu, São Paulo, Brazil; 2 Departamento de Patologia, Instituto Lauro de Souza Lima, Bauru, São Paulo, Brazil; 3 Departamento de Microbiologia, Instituto Lauro de Souza Lima, Bauru, São Paulo, Brazil; 4 Serviço Especializado em Dermatologia, Hospital das Clínicas do Acre, Rio Branco, São Paulo, Brazil; 5 Departamento de Ciências Biológicas, Faculdade de Odontologia de Bauru, Universidade de São Paulo, Bauru, São Paulo, Brazil; 6 Departamento de Ciências da Saúde, Universidade do Sagrado Coração, Bauru, São Paulo, Brazil; INSERM-Université Paris-Sud, FRANCE

## Abstract

Jorge Lobo’s disease (JLD) is a chronic infection that affects the skin and subcutaneous tissues. Its etiologic agent is the fungus *Lacazia loboi*. Lesions are classified as localized, multifocal, or disseminated, depending on their location. Early diagnosis and the surgical removal of lesions are the best therapeutic options currently available for JLD. The few studies that evaluate the immunological response of JLD patients show a predominance of Th2 response, as well as a high frequency of TGF-β and IL-10 positive cells in the lesions; however, the overall immunological status of the lesions in terms of their T cell phenotype has yet to be determined. Therefore, the objective of this study was to evaluate the pattern of Th1, Th2, Th17 and regulatory T cell (Treg) markers mRNA in JLD patients by means of real-time PCR. Biopsies of JLD lesions (N = 102) were classified according to their clinical and histopathological features and then analyzed using real-time PCR in order to determine the expression levels of TGF-β1, FoxP3, CTLA4, IKZF2, IL-10, T-bet, IFN-γ, GATA3, IL-4, IL-5, IL-13, IL-33, RORC, IL-17A, IL-17F, and IL-22 and to compare these levels to those of healthy control skin (N = 12). The results showed an increased expression of FoxP3, CTLA4, TGF-β1, IL-10, T-bet, IL-17F, and IL-17A in lesions, while GATA3 and IL-4 levels were found to be lower in diseased skin than in the control group. When the clinical forms were compared, TGF-β1 was found to be highly expressed in patients with a single localized lesion while IL-5 and IL-17A levels were higher in patients with multiple/disseminated lesions. These results demonstrate the occurrence of mixed T helper responses and suggest the dominance of regulatory T cell activity, which could inhibit Th-dependent protective responses to intracellular fungi such as *L*. *loboi*. Therefore, Tregs may play a key role in JLD pathogenesis.

## Introduction

Jorge Lobo's disease (JLD) is a chronic infection that affects mainly the skin and subcutaneous tissues. Its etiologic agent is the fungus *Lacazia loboi*. Evidence suggests that contamination by the fungus *L*. *loboi* often occurs as a result of skin trauma [1,2]; it most frequently affects the outer ear, the face, and the upper and lower limbs of rural workers [3,4]. JLD is endemic to the Amazon rainforest region [1,5,6]; out of the 322 cases reported in Brazil thus far, 249 cases have been from the state of Acre, which is located in the Amazon region [1,2]. Though it is endemic to this area [1,5,6], JLD has also been diagnosed in Central America, North America, Europe and South Africa [1,7,8,9], with approximately 550 cases reported worldwide [10].

Currently, JLD diagnosis is based on a clinical evaluation of the patient. This evaluation consists of a macroscopic examination of lesions, which may be keloid-like, verruciform and/or gummy; there may also be ulcerative lesions [10,11]. The fungus can also be detected in skin lesions via histopathological and mycological examinations [1]. The clinical forms of the disease are classified based on its distribution; the disease can be classified as localized (confined to a single area), multifocal (on a limb or limb segment), or disseminated (involving several anatomical regions) [12,13]. Histopathological analyses provide evidence of a granulomatous process, with intense diffuse histiocytic reaction, with large numbers of multinucleated giant cells, foreign body and/or Langhans cells [14], and with a cell infiltrate composed of few CD4 and CD8 T lymphocytes, NK cells, plasma cells and B lymphocytes [14]. In addition, numerous fungi have been observed in foreign-body giant cells and in the formation of syncytia [10].

Early diagnosis of JLD plays a key role in the treatment outcome, since there is currently no fully effective treatment available [5,10], particularly in cases of disseminated disease. The surgical excision of lesions is currently the best treatment option available, particularly for isolated lesions. Despite the common occurrence of relapses, [7,15,16,17], the removal of lesions provides a significant increase in patients’ quality of life [10].

It is well known that immune response plays an essential role in the outcome of fungal diseases [18,19]. The effectiveness of the antifungal response depends on the T cell subpopulations involved in the host’s response to fungi [18,19]. Th1 and Th17 responses are associated with effective protection against infection, during which the local production of IFN-γ (Th1 profile) and IL-17 (Th17 profile) stimulates antifungal effector functions of phagocytes, as well as the generation of optimal T-cell-dependent immunity [18,20]. Meanwhile, Th2 responses mediated by cytokines such as IL-4 and IL-13, as well as Treg cytokines such as IL-10 and TGF-β, can both result in the suppression of protective Th1 and Th17 responses. This suppression enables alternative macrophage activation and, as a consequence, makes fungal persistence more likely [18,19,20].

While the role of T helper subsets in antifungal responses is relatively well defined overall, there are still very few studies addressing the immunological aspects of JLD [12,21]. One study has suggested that the predominance of the Th2 profile facilitates the development of JLD based on the fact that patients’ peripheral blood mononuclear cells express high IL-4 levels when stimulated by *L*. *loboi* [22]. Additionally, an immunohistochemical analysis of the inflammatory infiltrate has revealed a high frequency of TGF-β1 and IL-10-positive cells in JLD patients’ lesions, as well as a slightly positive staining for TNF-α and iNOS [23,24]. While these findings were originally interpreted as additional evidence of a Th2 response in JLD lesions [23,24], the current knowledge allows us to hypothesize that the presence of Tregs may account for local TGF-β1 and IL-10 production and also points to the involvement of these T cell subsets in treatment outcomes for the lesions.

Given the scarcity of studies on the immunoregulatory mechanisms underlying JLD pathogenesis, the present study evaluated the cytokine expression profiles associated with Th1, Th2, Th17, and Treg cells in skin lesions and correlated them with the clinical forms of the disease (a single localized lesion and multiple lesions).

## Materials and Methods

### Sample Selection

The samples evaluated in this study were collected from patients treated in the Department of Specialized Dermatology at Acre Clinical Hospital in the city of Rio Branco, São Paulo State, Brazil, between 2008 and 2013. Samples were collected by a clinical team that also includes members of the Department of Pathology of the Lauro de Souza Lima Institute. The categorization of the JLD diagnosis as localized, multifocal, or disseminated was based on the clinical and histopathological features of the lesions. The inclusion criteria used were the presence of viable fungi in the histopathological exam and no recent history of specific antifungal treatment (“recent” was defined as “within the last year”). Lesion biopsies were taken at the moment of the diagnosis and stored in RNAlater solution for further analysis; they were added to a bank of biological samples kept at the Department of Pathology of the Lauro de Souza Lima Institute. Based on the similarity in number and aspect of multifocal lesions and disseminated lesions, the gene expression analysis used in this study combined multifocal and disseminated forms into a group hereby referred to as “patients with multiple lesions” (or “the multiple lesion group”), while the patients with the localized form of the disease were hereby referred to as “patients with a single lesion” (or “the single lesion group”). In addition, 12 biopsies from healthy individuals undergoing cosmetic skin surgery were used as the control group for this study.

Table 1 shows data on gender, age, and the duration of the lesion (JLD patients only). None of the JLD patients were under receiving immunosuppressive treatment at the time of their biopsy. Some patients (n = 62), however, were treated with itraconazole more than one year before the biopsies for this study were collected. The biopsies from patients with multiple lesions were collected from the most recent lesion, as indicated by the patient. Additionally, none of the patients had other kinds of skin lesions.

**Table 1:** Data on patients’ gender, age, and duration of lesions.

Group (number)	Gender(number)	Mean age (range)	Duration of lesion in years (± standard deviation)
Single (n = 54)	F (5)	44 (34–59)	14.6 (± 12.3)
	M (49)	49.2 (19–83)	16.1 (± 11.5)
Multiple (n-48)	F (5)	49 (38–56)	23.2 (± 16.7)
	M (43)	58.3 (21–86)	20.4 (± 12.7)
Control (n = 12)	F (6)	37 (23–50)	-
	M (6)	37 (21–59)	-

F: female, M: male.

### Ethics Statement

All procedures were carried out after the confirmation of approval from the Scientific and Research Ethics Committees of the Lauro de Souza Lima Institute under file numbers 251/13 and 368005. Written informed consent was obtained from all participants.

### Fungal Cell Counts

The histopathological slides used to select the samples were also used to estimate the number of fungal cells in the lesions. Two independent examiners (CTS and AFFB) observed the slides stained by hematoxylin and eosin. Five microscopic fields (100X amplification) were chosen randomly from the infiltrate area and examined. Viable fungal cells were those with intact cell walls and homogeneous internal cytoplasm content.

### RNA Extraction, Quality/Integrity Analysis, and cDNA Synthesis

The biopsies that had been stored in RNAlater were each cut into small fragments with a scalpel and transferred into tubes containing ceramic beads (CK28—Bertin Technologies). Then, 700μl of QIAzol reagent (Qiagen) was added in order to homogenize and lyse the samples in the Precellys24 system (Bertin Technologies). The system was run at one pulse (6500rpm) for 10 seconds, followed by 5 minutes of incubation at 4°C. The cycle was repeated 3 times.

Total RNA was extracted using the QIAGEN miRNeasy Mini Kit and the QIAcube robotic workstation according to the manufacturer’s instructions and was recovered in 30μl of ultrapure water. The quantification and purity (ratio 260/280) of the samples were evaluated in a NanoDrop 2000 spectrophotometer (Thermo Fisher Scientific), and sample ratios equal to or close to 2 were considered appropriate. Sample integrity was evaluated using the 2100 Bioanalyzer system (GE Healthcare Bio-Sciences) and the Agilent RNA 6000 Nano kit. Appropriate RNA samples were defined as those with Integrity Number (RIN) greater than or equal to 5.

Complementary DNA (cDNA) was synthesized by a reverse transcription reaction using the QuantiTect Reverse Transcription kit (QIAGEN) with 1μg RNA according to the manufacturer's instructions.

### Real-time PCR Reactions

For each of the selected targets, standard curves of the genes were prepared in triplicate using a pool of 8 samples (2 samples of each form, including healthy controls) and were diluted from 1:2 to 1:10 with 5 or 6 points beginning at concentrations of 50ng or 100ng. The genes were Treg [TGF-β1 (Hs00998133_m1), FoxP3 (Hs01085834_m1), CTLA4 (Hs03044418_m1), IL-10 (Hs00961622_m1) and IKZF2 (Hs00212361_m1)], Th1 [T-bet (TBX21-Hs00203436_m1) and IFN-γ (Hs00989291_m1)], Th2 [GATA3 (Hs00231122_m1), IL-5 (Hs01548712_g1), IL-4 (Hs00174122_m1), IL-13 (Hs00174379_m1) and IL-33 (Hs00369211_m1)], and Th17 [RORC—transcription human RORγt (Hs01076122_m1), IL-17A (Hs00174383_m1), IL-17F (Hs00369400_m1) and IL22 (Hs01574154_m1)]. In order to choose the best dilution level for the samples, the following parameters were considered: C_T_ variation of less than 0.5 among the triplicates, R^2^ greater than 0.9, efficiency between 95% and 105%, and slope close or equal to 3.3, as suggested in the MIQE Guidelines [25].

All of the cDNA samples were used in the real-time PCR reactions in order to detect mRNA for GAPDH (Hs03929097_g1), a constitutive expression gene (endogenous control) used both to check cDNA quality and for standardization (ΔC_T_). Assays were performed in duplicate in the ViiA™ 7 Real-Time PCR System (Applied Biosystems) using the TaqMan™ Gene Expression Master Mix and the TaqMan™ Gene Expression Assays as reagents, as determined by the manufacturer (Life Technologies). The reaction mix consisted of 5μL of Taqman™ Gene Expression Master Mix (10x), 0.5μL of Taqman™ Gene Expression Assay (20x), 3.5μL of ultrapure H_2_O (DNase and RNase free), and 1μl of the cDNA sample. The reactions were carried out as follows: 2 minutes at 50°C, 10 minutes at 95°C, 40 cycles of 15 seconds at 95°C, and 1 minute at 60°C (annealing temperature). Reactions were performed in duplicate in specific real-time PCR 96-well plates.

Once GAPDH positivity was confirmed, the expression of mRNA-encoding cytokines and the transcription factors characteristic of the Treg profiles (TGF-β1, FoxP3, CTLA4, IKZF2 and IL-10), Th1 (T-bet and IFN-γ), Th2 (GATA3, IL-5, IL-4, IL-13 and IL-33) and Th17 (RORC, IL-17A, IL-17F and IL-22) were also analyzed by real-time PCR reactions using the TaqMan™ Gene Expression Assays, which had been previously standardized by the manufacturer (Life Technologies) and which contained primers and probes specific to each target. Results are expressed as ΔC_T_, in which low ΔC_T_ reflects high expression of the target and high ΔC_T_ reflects low expression of the target.

### Data Analysis

Statistical analysis of the ΔC_T_ values (C_T_ GAPDH value—C_T_ target value) were carried out using the Mann-Whitney U test within the GraphPad Prism 5.0 software (GraphPad). Additionally, the linear regression test was used to correlate ΔC_T_ values with the total number of fungal cells, the number of viable fungal cells, and the duration of the lesions.

## Results

### Samples

A total of 114 samples were used in the gene expression analysis. Of these 114 samples, 54 corresponded to the localized form, 20 corresponded to the multifocal form, and 28 corresponded to the disseminated form. Twelve healthy controls were also used to evaluate gene expression. Therefore, there were a total of 48 patients with multiple lesions 54 patients with a single lesion.

### Gene Expression

**Fig 1:**
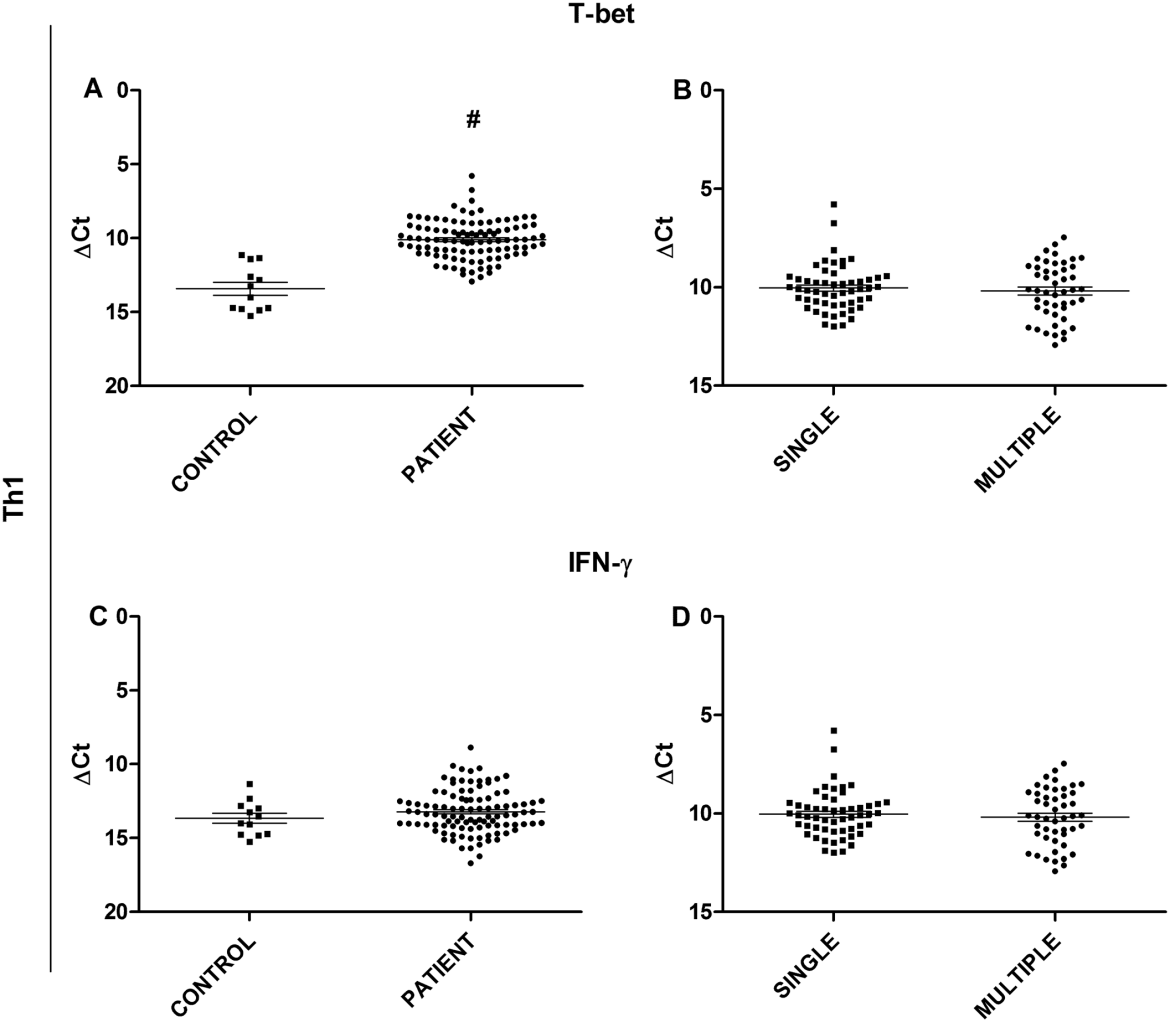
Gene expression analysis of Th1 profile targets. (A) Comparison of T-bet transcription factor expression between JLD patients and healthy controls. (B) Comparison of T-bet transcription factor expression between patients with single lesion and multiple lesions. (C) Comparison of IFN-γ cytokine expression between JLD patients and healthy controls. (D) Comparison of IFN-γ cytokine expression between patients with a single lesion and patients with multiple lesions. Statistical analysis: Mann-Whitney U test (#p≤0.05)

The analysis also considered the expression of T-bet and IFN-γ, both of which are Th1 profile targets. T-bet transcription factor (Fig 1A) expression was significantly higher in the patients than in the controls (p<0.0001). However, the analysis of IFN-γ mRNA levels (Fig 1C) revealed no significant difference between the two groups. Additionally, no significant difference between the single or multiple forms was observed for either of the targets (Fig 1B and 1D).

**Fig 2:**
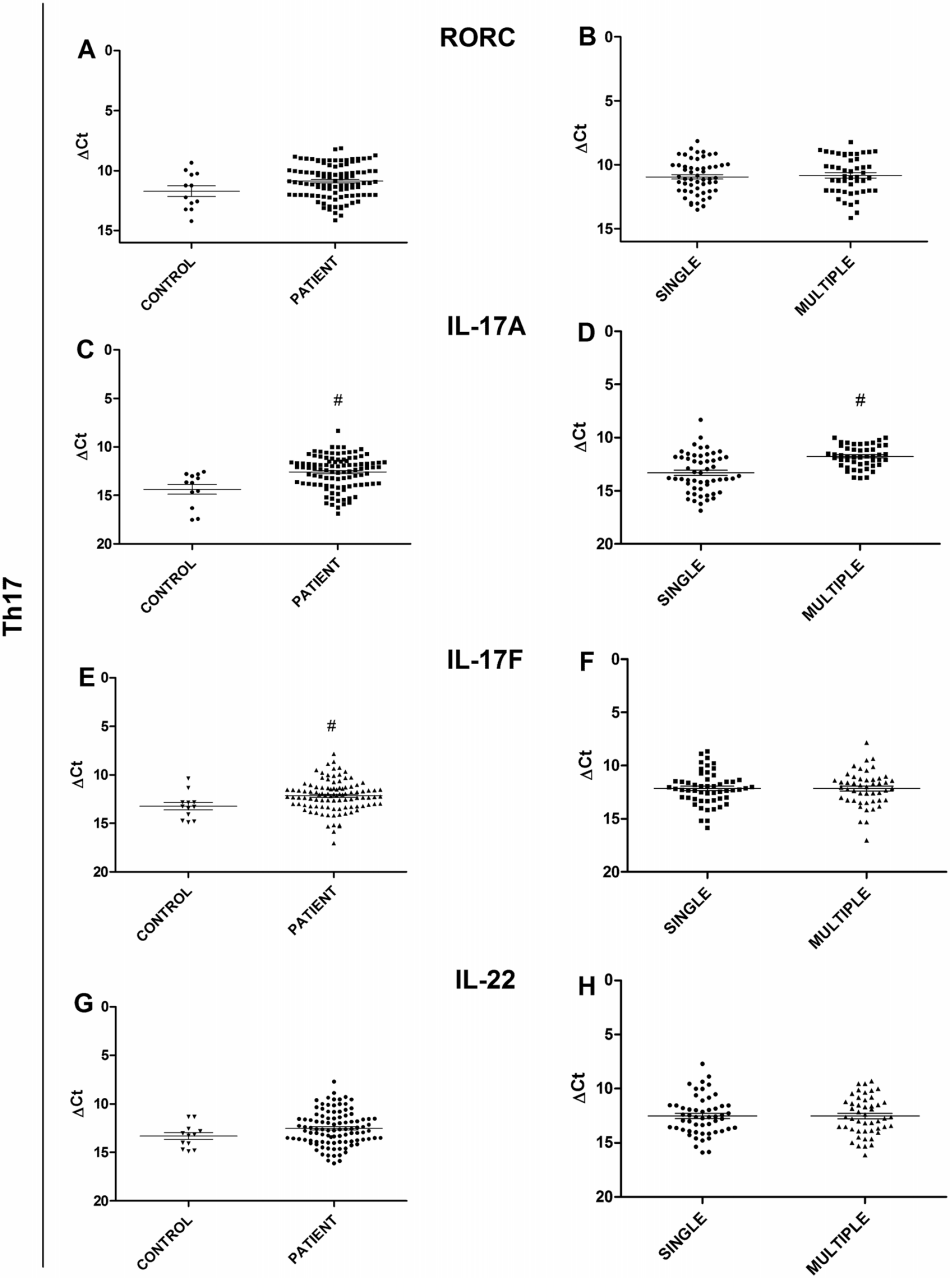
Gene expression analysis of Th17 profile targets. (A) Comparison of RORC transcription factor between JLD patients and healthy controls. (B) Comparison of RORC transcription factor between patients with a single lesion and patients with multiple lesions. (C) Comparison of IL-17A cytokine expression between JLD patients and healthy controls. (D) Comparison of IL-17A cytokine expression between patients with a single lesion and patients with multiple lesions. (E) Comparison of IL-17F cytokine expression between JLD patients and healthy controls. (F) Comparison of IL-17F cytokine expression between patients with a single lesion and patients with multiple lesions. (G) Comparison of IL-22 cytokine expression between JLD patients and healthy controls. (H) Comparison of IL-22 cytokine expression between patients with single lesion and patients with multiple lesions. Statistical analysis: Mann-Whitney U test (#p≤0.05)

The selected targets of the Th17 profile were RORC, IL-17A, IL-17F, and IL-22. The expression of IL-17A and IL-17F cytokines was found to be significantly higher (p = 0.0025, p = 0.0168, respectively) in patients when compared the controls (Fig 2C and 2E), and the expression of IL-17A (Fig 2D) was significantly higher in patients with multiple lesions than it was in patients with a single lesion (p<0.0001). However, the expression of IL-17F did differ significantly between the single and multiple forms (Fig 2F). When the expression of RORC and IL-22 cytokines was analyzed, no significant difference was found between the patients and the controls, nor between the single lesion group and the multiple lesion group (Fig 2A, 2B, 2G and 2H).

**Fig 3:**
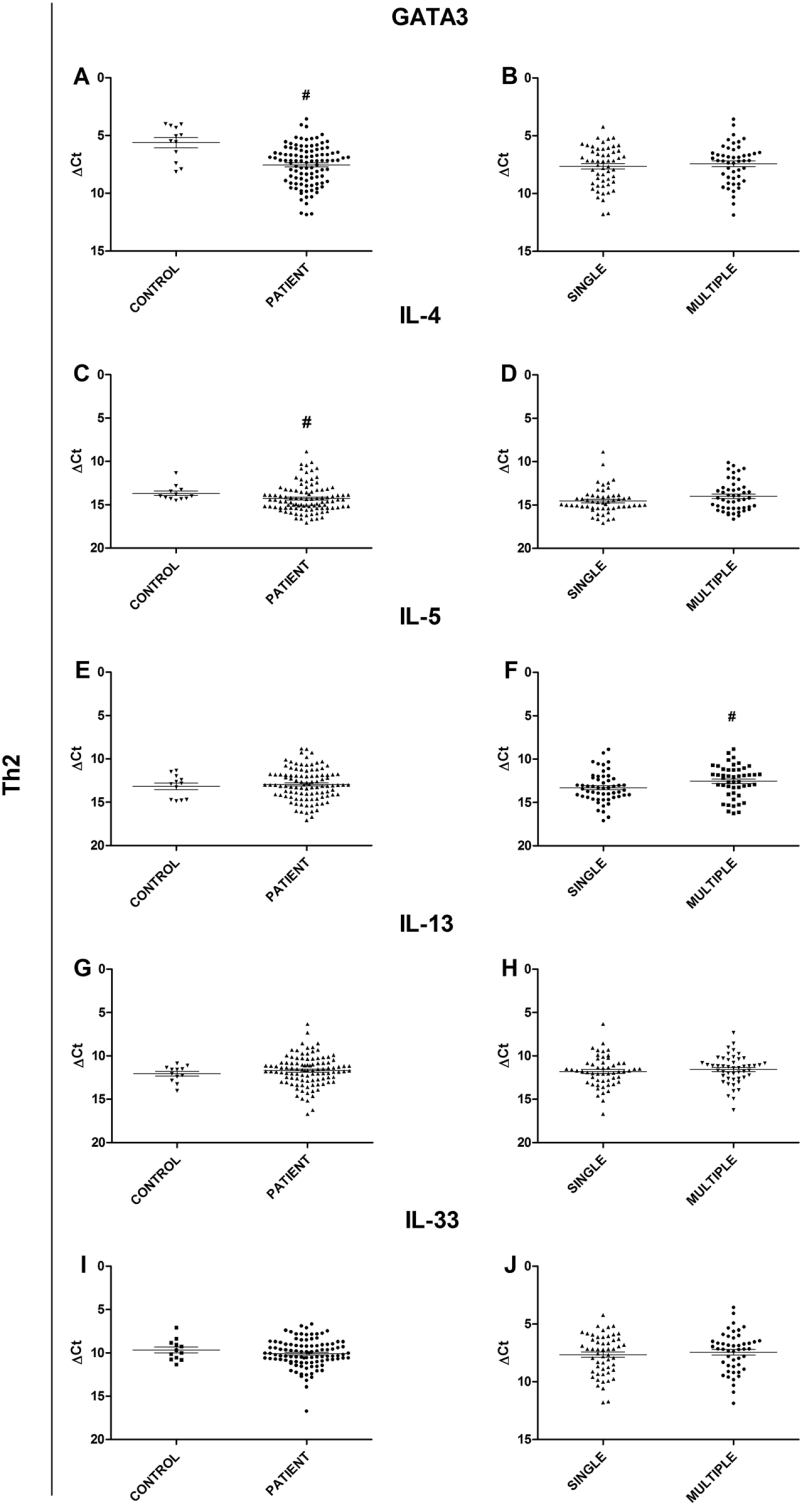
Gene expression analysis of Th2 profile targets. (A) Comparison of the GATA3 transcription factor between JLD patients and healthy controls. (B) Comparison of GATA3 transcription factor between patients with a single lesion and patients with multiple lesions. (C) Comparison of IL-4 cytokine expression between JLD patients and healthy controls. (D) Comparison of IL-4 cytokine expression between patients with a single lesion and patients with multiple lesions. (E) Comparison of IL-5 cytokine between JLD patients and healthy controls. (F) Comparison of IL-5 cytokine expression between patients with a single lesion and patients with multiple lesions. (G) Comparison of IL-13 cytokine expression between JLD patients and healthy controls. (H) Comparison of IL-13 cytokine expression between patients with a single lesion and patients with multiple lesions. (I) Comparison of IL-33 cytokine expression between JLD patients and healthy controls. (J) Comparison of IL-33 cytokine expression between patients with a single lesion and patients with multiple lesions. Statistical analysis: Mann-Whitney U test (#p≤ 0.05)

When the Th2 profile targets were analyzed, the GATA3 transcription factor (p = 0.0089) and the cytokine IL-4 (p = 0.0399) presented significantly lower expression in the patients than in the controls (Fig 3A and 3C), whereas IL-5, IL-13, and IL-33 levels did not differ significantly between the patients and the controls (Fig 3E, 3G and 3I). Similarly, no significant differences in IL-13 levels or IL-33 levels (Fig 3H and 3J) were observed between the single lesion group and the multiple lesion group. However, expression of IL-5 (Fig 3F) was found to be significantly higher in the multiple lesion group than in the single lesion group (p = 0.0144).

**Fig 4:**
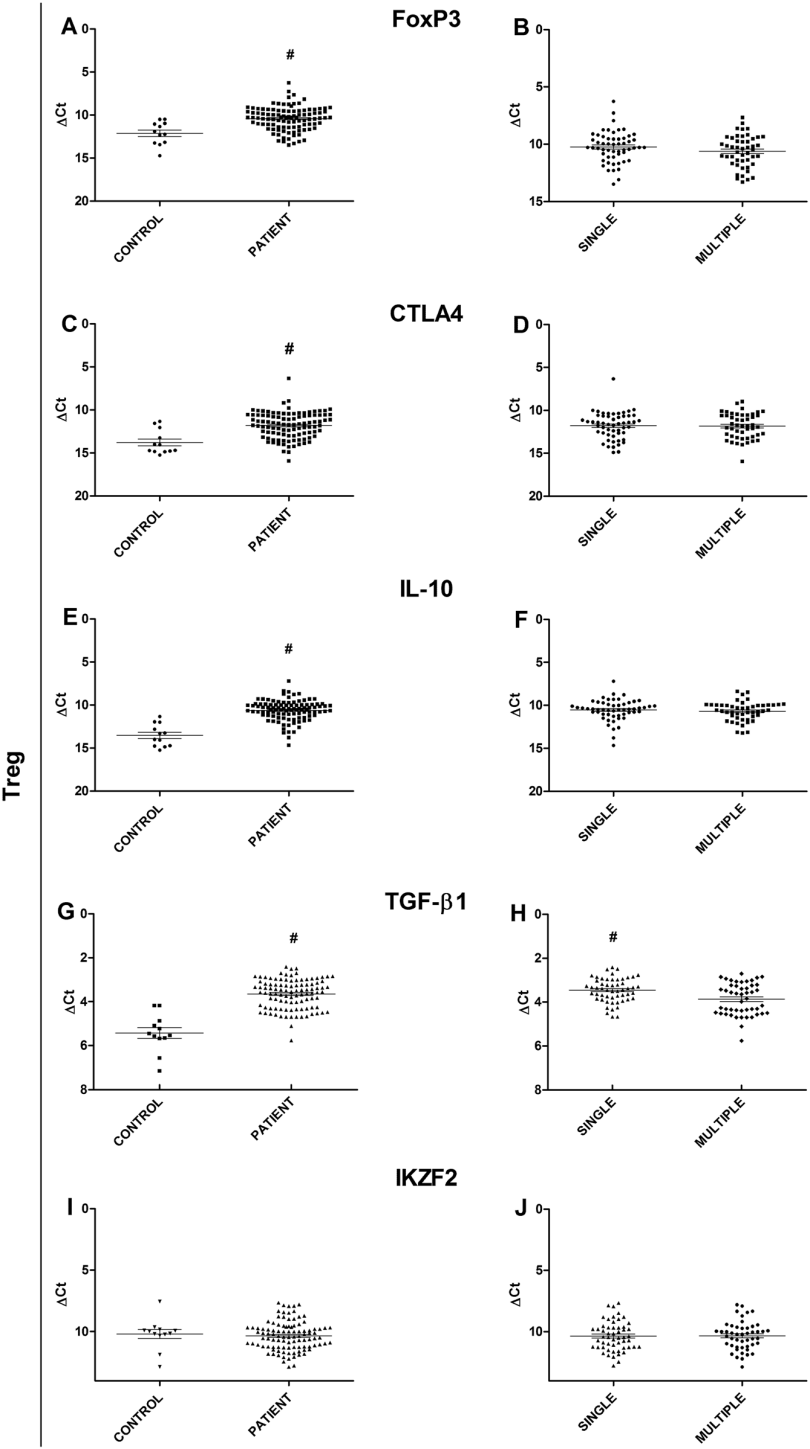
Gene expression analysis of Treg profile targets. (A) Comparison of transcription factor FoxP3 between JLD patients and healthy controls. (B) Comparison of FoxP3 transcription factor between patients with a single lesion and patients with multiple lesions. (C) Comparison of CTLA4 marker expression between JLD patients and healthy controls. (D) Comparison of CTLA4 marker expression between patients with a single lesion and patients with multiple lesions. (E) Comparison of IL-10 cytokine expression between JLD patients and healthy controls. (F) Comparison of IL-10 cytokine expression between patients with a single lesion and patients with multiple lesions. (G) Comparison of TGF-β1 cytokine expression between JLD patients and healthy controls. (H) Comparison of TGF-x1 cytokine expression between patients with a single lesion and patients with multiple lesions. (I) Comparison of IKZF2 marker expression between JLD patients and healthy controls. (J) Comparison of IKZF2 marker expression between patients with a single lesion and patients with multiple lesions. Statistical analysis: Mann-Whitney U test (#p≤0.05)

The expression of specific Treg profile targets were also considered. TGF-β1 (p<0.0001), IL-10 (p<0.0001), CTLA4 (p = 0.0018), and FoxP3 (p = 0.0025) expression levels were found to be significantly higher in lesions from patients than in healthy skin controls (Fig 4A, 4C, 4E and 4G). Conversely, the analysis of IKZF2 mRNA levels showed no significant difference between the patients with lesions and the controls (Fig 4I). In addition, when the expression of Treg markers were compared between patients with multiple lesions and patients with a single lesion, TGF-β1 (Fig 4H) expression levels were found to be significantly higher in patients from the single lesion group (p = 0.0059). The other targets were evaluated (IKZF2, IL-10, FoxP3 and CTLA4), but there was no significant difference when the single lesion group was compared to the multiple lesion group (Fig 4B, 4D, 4F and 4J).

### Correlation Analysis

**Fig 5:**
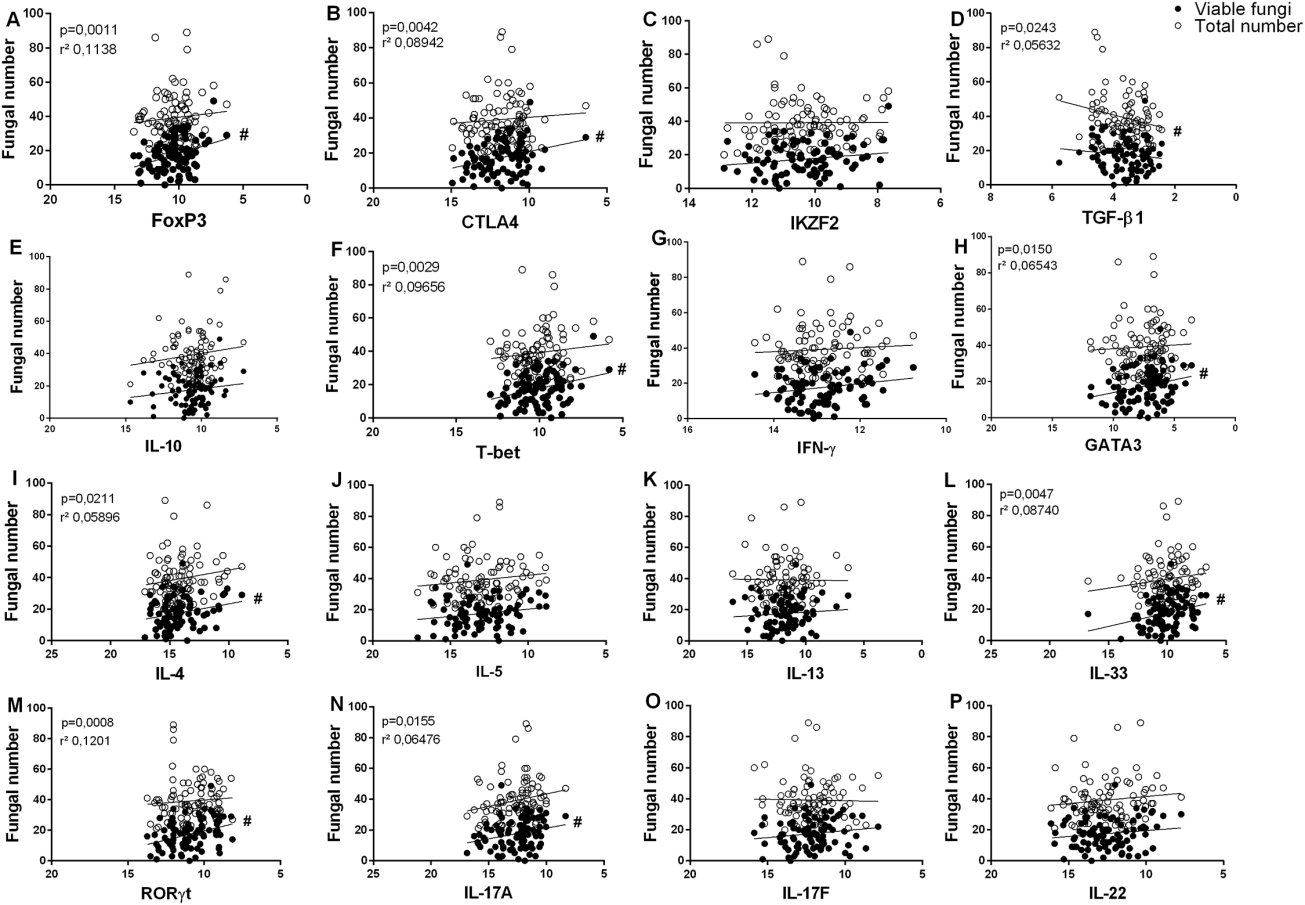
Linear regression and significance among Th1, Th2, Th17 and Treg profile targets based on ΔC_T_ values, total number of fungal cells, and number of viable fungal cells. Analysis of T cell subsets: Treg (A to E), Th1 (F and G), Th2 (H to L), Th17 (M to P). **#**: in front of the regression lines represents significant correlation (p and r^2^ values). Regression analysis was performed using patient data.

Fig 5 shows the negative correlation found between ΔC_T_ values (FoxP3, CTLA4, T-bet, GATA3, IL-4, IL-33, RORC, and IL-17A) and the number of viable fungal cells. The total number of fungal cells was found to correlate significantly only with TGF-β1. Note that, because values were expressed as ΔC_T_, low ΔC_T_ reflects high expression of the target. No correlation was observed between ΔC_T_ values and the duration of the lesions (data not shown).

## Discussion

While few previous studies report a dominance of immunosuppressive cytokines in the host response triggered by *L*. *loboi* infection [14], the exact nature of the host response in the immunopathogenesis of JLD remains to be established, particularly in terms of the T helper subsets.

Though the specific mechanisms involved in eliminating *L*. *loboi* have not been determined, the body’s protective response to fungi is generally known to involve Th1 and/or Th17 responses, which mediate the chemoattraction of phagocytes and their subsequent activation, thus enabling fungal elimination [18,26].

While our results demonstrate an increase in the levels of T-bet, a transcription factor responsible for Th1 cell polarization, the levels of the prototypical Th1 cytokine IFN-γ did not increase in the JLD lesions. Considering the fact that *L*. *loboi* is an intracellular organism [9,27,28], IFN-γ may play a critical role in the elimination of *L*. *loboi* through the activation of macrophages, as described in studies on other intracellular pathogens [18,29]. Therefore, it is possible to hypothesize that the absence of high levels of IFN-γ may contribute to the persistence of *L*. *loboi* that is characteristically observed in JLD patients’ lesions.

Th17 cell contribution to antifungal response is still debated. Some studies show that Th17 cells play a protective role in the control of fungal infections [26,30,31], while other studies associate the Th17 response to the organism’s survival and multiplication [32,33,34]. Our results show that both IL-17A expression and IL-17F expression are higher in the lesions than in the controls, suggesting that the development of the Th17 response may not provide effective protection against *L*. *loboi* infection. Therefore, while IL-17 may contribute to the recruitment of monocytes to the lesion site, which in theory could provide some antifungal protection [35], the absence of a robust local Th1 response (represented by the modest IFN-γ levels) may result in fungal persistence.

However, it is also possible to argue that, despite the statistically significant increase in IL-17A and IL-17F, from a biological standpoint such a limited increase it may not be representative of a robust Th17 response. In theory, this limitation could enable the elimination of the fungus from the lesion sites. In accordance with this hypothesis, Th17 responses are typically associated with significant neutrophil recruitment [36,37], but neutrophils are not frequently found in JLD lesions. In addition, other Th17-related cytokines, such as IL-22 [38], were not upregulated in the lesions, a finding which supports the idea that robust Th17 responses do not take place in JLD lesions.

Interestingly, previous studies have found that enhanced migration and the actions of Tregs at sites of infection usually benefit the pathogen by disabling both Th1 and Th17 responses [39]. We found Treg markers (FoxP3, CTLA-4, IL-10 and TGF-β1) to also be expressed in normal control skin, a finding which is consistent with previous reports [40,41]. Indeed, normal skin is described as an important site of Treg induction and residence. In fact, the presence of Tregs as 5% to 10% of the resident T cells in normal human skin contributes to the maintenance of the homeostasis of this environment [40]. When the expression of Treg markers in lesions and in controls was compared, the data demonstrated that FoxP3, CTLA-4, IL-10, and TGF-β1 levels were higher in the patients’ lesions than in the control group. A similar pattern has been described in a study on cutaneous lesions from patients with paracoccidioidomycosis [42]. In paracoccidioidomycosis, Tregs act as active immunosuppressive cells [39,43], presenting a dominant role over other Th subsets. This results in impaired infection control [39,43,44,45]. A similar situation has been described in studies on other fungal infections, in which the Tregs’ suppression of the host response is a key element in fungal persistence [39,43,44,45].

As previously mentioned, Treg markers (i.e. FoxP3, CTLA-4, IL-10 and TGF-β) were found to be upregulated in the JLD lesions to a higher extent than the slight increase seen in IL-17 levels. This result contributes to the hypothesis that Tregs could play a dominant role in the lesion environment. This hypothesis is based only on associative data, however, and further functional analysis would be required to support it.

Previous studies that have focused on the immunopathogenesis of fungal infections provide some parallel support. For example, Treg products widely expressed in JLD lesions, such as TGF-β1 and IL-10, are able to inhibit IFN-γ synthesis [46], which could account for the poor IFN-γ expression in the lesions. Indeed, impaired Th1 response due the presence and activation of Tregs was found to directly interfere in the outcome fungal infections [39,47]. Also, Tregs and their products can limit the polarization and effector function of Th17 cells and the development of robust Th17 responses [48]. These factors could account for the limited expression of Th17-markers in the lesions. While macrophages and fibroblasts may be responsible for IL-17 expression in the lesions [49,50,51], the putative inhibitory effect that Tregs have on Th17 cells may be critical in preventing the development of comprehensive Th17 responses in the lesions. A recent study demonstrated that IL-17+ cells outnumbered FOXP3+ cells in JLD lesions [52]. This report is consistent with the findings regarding IL-17 and FOXP3 expression in the present study. However, the previous authors concluded that high levels of Th17 cytokines could overcome the effects of Treg cells [52]. This assertion does not consider the fact that Tregs make up a very small fraction of lymphocyte subsets both, in the circulating lymphocytes pool and in inflammatory infiltrates in peripheral tissues; it is not surprising that effector cells, such as IL-17+ cells, may outnumber FOXP3+ cells [53,54]. Indeed, Tregs are thought to present a dominant role in inflammatory infiltrates even as an outnumbered population relative to the number of effector cells [53,54]. Therefore, the simple analysis of IL-17+/FOXP3+ proportions in the lesions without comparisons to control samples and/or to other lesion forms or stages is not necessarily informative of the true T cell balance.

It is important to keep in mind that the interaction between Treg cells and Th17 cells in fungal diseases is still disputed [55]. In the case of candidiasis, for example, Treg cells can both inhibit [56] or promote [30,57] Th17 response.

While Th1 and Th17 responses may contribute to the control of fungal infections, Th2 responses have been described as playing an opposing role. Interestingly, before the identification of Tregs and Th17, Th2 cells were originally described as suppressors of Th1 antifungal activity in the original Th1-Th2 archetype [26,58]. In the traditional immune response to fungi, Th2 responses were thought to be harmful to the host, as they have been associated with increased fungal burden [26,59,60]. Th2 cytokines such as IL-4 and IL-13 inhibit Th1 response and induce alternative macrophage activation, thus resulting in an anti-inflammatory process [61] that would favor the multiplication of *L*. *loboi*. Interestingly, while no differences in the levels of Th2 factors IL-5, IL-13, or IL-33 were observed between the JLD patients and the control group, the levels of IL-4 and GATA3 transcription factor were lower among patients. This information is in line with the hypothesis of a dominance of Tregs in the JLD lesions; this cell type suppresses not only the function of Th1 and Th17 cells, as previously discussed, but also the function of the Th2 subset [39,62], which could account for the limited local expression of Th2 markers. Accordingly, in experimental models of fungal infection, Tregs were previously described to simultaneously inhibit all of the Th subsets, resulting in impaired antifungal mechanisms and more severe tissue damage [39]. Indeed, the effectiveness of the antifungal responses clearly involved multiple Th subsets types, and the combined actions of the Th1 and Th17 subsets seem to be important in the protection against fungal diseases [18,63].

This study compared the expression of targets in a control group to targets in patients and also evaluated the possible variation in the expression profiles of Th markers by comparing single and multiple forms of JLD. Patients with multiple and disseminated forms of JLD exhibit clinical similarities, and multiple forms often progress to disseminated disease. Therefore, for the purpose of immune response analysis, these two forms were grouped together and compared to the single form of the disease, as this form usually does not progress to other forms. In this analysis, TGF-β1 presented a higher expression in patients with a single lesion while IL-17A and IL-5 levels were significantly higher in patients with multiple lesions.

Higher TGF-β1 expression in the single lesion group was unexpected; because this cytokine is associated with immunosuppression, higher levels were expected to be detected in the multiple lesion group. Nevertheless, in this case, TGF-β1 could be associated with the fibrotic status of the lesions [64,65], and this process should be more evident in the single form of JLD, favoring the containment of the granulomas. Additional studies evaluating collagen fibers are needed to clarify this hypothesis.

Meanwhile, IL-5 cytokine expression did not differ between controls and patients; however, this expression was higher in the multiple lesion group, a result which indicates that this cytokine may influence the dissemination of the disease. The role of the IL-5 cytokine, which is associated with eosinophil recruitment and survival, has also been associated with susceptibility to lung infection with *C*. *neoformans* [66]. Furthermore, it has been recently shown that eosinophils produce IL-4 and therefore contribute to the development of the Th2 response, which is not considered protective in cases of fungal infections [67]. Nevertheless, eosinophils are scarce in JLD lesions and are unlikely to play a prominent role.

As mentioned previously, the higher IL-17A expression in the multiple lesion groups suggests that, despite a statistically significant variation in IL-17 levels, there is no robust local Th17 response. This finding reinforces the hypothesis that the Th17 response does not provide effective protection against *L*. *loboi* infection, a result which is likely due to the intracellular characteristics of this fungus.

The correlation between viable fungal burden and target expression was negative for at least one marker for each immune response profile; therefore, the results did not allow for the definition of a standard response that could be responsible for disease outcome. Additionally, there were no correlations found between target expression and the total number of fungi or between target expression and the duration of the lesion. This process may occur because, though lesions often last for long periods of time, their progression is slow and continuous. In the present study, all of the targets that were expressed were found in newly developed lesions from chronically diseases patients.

In conclusion, our results suggest that the local overexpression of IL-10, TGF-β1, and CTLA-4 in JLD lesions may be indicative of a dominance of Tregs in the lesions. This hypothesis is reinforced by the limited expression of Th effector cell markers in the lesions.

Assuming that protective immunity against intracellular fungi such as *L*. *loboi* depends on adequate Th1 and Th17 responses, the putative dominance of Treg activity may play a significant role in both fungal survival and the formation of lesions. However, functional studies are certainly required to confirm the possibility of Treg-mediated immunoregulation in the lesions. The understanding of the immunoregulation involved in JLD can contribute to new diagnostic and therapeutic approaches that will ultimately lead to better disease management.

## References

[1] Brito AC, Quaresma JAS. Lacaziose (doença de Jorge Lobo): revisão e atualização. An Bras Dermatol. 2007;82:461–74.

[2] Woods WJ, Belone AF, Carneiro LB, Rosa PS. Ten years experience with Jorge Lobo’s disease in the state of Acre. Brazil. Rev Inst Med Trop São Paulo. 2010;52:273–8. 21049233 10.1590/s0036-46652010000500010

[3] Herr RA, Tarcha EJ, Taborda PR, Taylor JW, Ajello L, Mendoza L. Phylogenetic analysis of *Lacazia loboi* places this previously uncharacterized pathogen within the dimorphic *Onygenales* . J Clin Microbiol. 2001;39:309–14. 11136789 10.1128/JCM.39.1.309-314.2001 PMC87720

[4] Paniz-Mondolfi AE, Reyes-Jaimes O, Dávila-Jones L. Lobomycosis in Venezuela. Int J Dermatol. 2007;46:180–5. 17269972 10.1111/j.1365-4632.2007.02937.x

[5] Lacaz CS, Baruzzi RG, Rosa MCB. Doença de Jorge Lobo. São Paulo: USP-IPSIS;1986.

[6] Rodríguez-Toro G. Lobomycosis. Int J Dermatol. 1993;32(5):325–32. 10.1111/j.1365-4362.1993.tb01466.x 8505156

[7] Talhari S, Talhari C. Lobomycosis. Clin Dermatol. 2012;30:420–4. 10.1016/j.clindermatol.2011.09.014 22682191

[8] Papadavid E, Dalamaga M, Kapniari I, Pantelidaki E, Papageorgiou S, Pappa V, et al Lobomycosis: A case from Southeastern Europe and review of the literature. J Dermatol Case Rep. 2012;3:65–9. 10.3315/jdcr.2012.1104 PMC3470791 23091581

[9] Arju R, Kothadia JP, Kaminski M, Abraham S, Giashuddin S. Jorge Lobo’s disease: a case of keloidalblastomycosis (lobomycosis) in a nonendemic area. Ther Adv Infect Dis. 2014;2(3–4):91–96 10.1177/2049936114559919 25469235 PMC4250272

[10] Paniz-Mondolfi A, Talhari C, Hoffmann LS, Connor DL, Talhari S, Bermudez-Villapol L, et al Lobomycosis: an emerging disease in humans and delphinidae. Mycoses 2012;55:298–309. 10.1111/j.1439-0507.2012.02184.x 22429689

[11] Tubilla LHM, Schettini APM, Eiras JC, Graça CZA, Frota MZM. Lacaziose simulando hanseníase dimorfa tuberculóide. An Bras Dermatol 2008;83(3):261–263.

[12] Opromolla DVA, Taborda PROW, Taborda VBA, Viana S, Furtado JF. Lobomicose: relato de 40 casos novos. An Bras Dermatol 1999;74:135–141.

[13] Opromolla DVA, Belone AFF, Taborda PRO, Taborda VBA. Correlação clinicopatológica em 40 casos novos de lobomicose. An bras Dermatol 2000;75(4):425–434.

[14] Vilani-Moreno FR, Belone AFF, Soares CT, Opromolla DVA. Immunohistochemical characterization of the cellular infiltrate in Jorge Lobo’s disease. Rev Iberoam Micol 2005;22:44–49. 15813683 10.1016/s1130-1406(05)70006-1

[15] Wiersema JP, Niemel PLA. Lobo's disease in Surinan patients. Trop Geogr Med. 1965;17:89–111. 5853472

[16] Restrepo A. Treatment of tropical mycoses. J Am Acad Dermatol. 1994;31(3 Pt 2):S91–S102. 8077517 10.1016/s0190-9622(08)81277-7

[17] Miranda MFR, Costa VS, Bittencourt MJS, Brito AC. Transepidermal elimination of parasites in Jorge Lobo’s disease. An Bras Dermatol. 2010;86(2):39–43. 10.1590/s0365-05962010000100005 20464085

[18] Borghi M, Renga G, Puccetti M, Oikonomou V, Palmieri M, Galosi C, et al Antifungal Th Immunity: Growing up in Family. Front Immunol. 2014 10 15;5:506 10.3389/fimmu.2014.00506 eCollection 2014. 25360137 PMC4197763

[19] Verma A, Wüthrich M, Deepe G, Klein B. Adaptive immunity to fungi. Cold Spring Harb Perspect Med. 2014 11 6;5(3):a019612 10.1101/cshperspect.a019612 25377140 PMC4355251

[20] Abbas AK, Lichtman AH, Pillai S. Imunologia celular e molecular 7a ed Rio de Janeiro:Elvieser; 2012.

[21] Marcos EVC, Souza FC, Torres EA, Lauris JRP, Opromolla DVA. Estudo da associação entre antígenos leucocitários humanos e doença de Jorge Lobo. Rev Soc Bras de Med Tropical. 2005;38(5):399–401. 10.1590/s0037-86822005000500007 16172755

[22] Vilani-Moreno FR, Lauris JR, Opromolla DVA. Cytokine quantification in the supernatant of mononuclear cell cultures and in blood serum from patients with Jorge Lobo's disease. Mycopathologia. 2004;158(1):17–24. 15487315 10.1023/b:myco.0000038433.76437.ec

[23] Xavier MB, Ferreira MMR, Quaresma JAS, Brito AC. HIV and lacaziosis, Brazil. Emerg Infect Dis. 2006;12(3):526–7. 16710984 10.3201/eid1203.051426 PMC3291465

[24] Vilani-Moreno FR, Belone AFF, Lara VS, Venturini J, Lauris JRP, Soares CT. Detection of cytokines and nitric oxide synthase in skin lesions of Jorge Lobo’s disease patients. Med Mycol. 2011;Early Online:1–6. 21208026 10.3109/13693786.2010.547993

[25] Bustin SA, Benes V, Garson JA, Hellemans J, Huggett J, Kubista M, et al The MIQE guidelines: minimum information for publication of quantitative real-time PCR experiments. Clin Chem. 2009 4;55(4):611–22. 10.1373/clinchem.2008.112797 Epub 2009 Feb 26. 19246619

[26] Romani L. Immunity to fungal infection. Nat Rev Immunol. 2011;11(4):275–88. 10.1038/nri2939 21394104

[27] Burns RA, Roy JS, Woods C, Padhye AA, Warnock D. Report of the first human case of lobomycosis in the United States. J Clin Microbiol. 2000;38:1283–5. 10699043 10.1128/jcm.38.3.1283-1285.2000 PMC88608

[28] Rosa PS, Soares CT, Belone AFF, Vilela R, Ura S, Filho MC, et al Accidental Jorge Lobo’s disease in a worker dealing with Lacazia loboi infected mice: a case report. Journal of Medical Case Reports. 2009;3:67 10.1186/1752-1947-3-67 19220901 PMC2647936

[29] Muraille E, Leo O, Moser M.TH1/TH2 paradigm extended: macrophage polarization as an unappreciated pathogen-driven escape mechanism? Front Immunol. 2014 11 26;5:603 10.3389/fimmu.2014.00603 25505468 PMC4244692

[30] Huang W, Na L, Fidel PL, Schwarzenberger P. Requirement of interleukin-17A for systemic anti-Candida albicans host defense in mice. J. Infect. Dis. 2004; 190:624–631. 15243941 10.1086/422329

[31] Espinosa V, Rivera A. Cytokines and the regulation of fungus-specific CD4 T cell differentiation. Cytokine 2012; 58: 100–106. 10.1016/j.cyto.2011.11.005 22133343 PMC3290683

[32] Zelante T, De Luca A, Bonifazi P, Montagnoli C, Bozza S, Moretti S, et al IL-23 and the Th17 pathway promote inflammation and impair antifungal immune resistance. Eur. J. Immunol. 2007 37: 2695–2706.8 17899546 10.1002/eji.200737409

[33] Koh AY, Kohler JR, Coggshall KT, Van Rooijen N, Pier GB. Mucosal damage and neutropenia are required for Candida albicans dissemination. PLoS Pathog. 2008 4: e359 10.1371/journal.ppat.0040035 18282097 PMC2242836

[34] MacCallum DM, Castillo L, Brown AJ, Gow NA, Odds FC. Early-expressed chemokines predict kidney immunopathology in experimental disseminated Candida albicans infections. PLoS One. 2009;4:e6420 10.1371/journal.pone.0006420 19641609 PMC2712765

[35] Shahrara S, Pickens SR, Mandelin AM, Karpus WJ, Huang Q, Kolls JK, et al IL-17-mediated monocyte migration occurs partially through CC chemokine ligand 2/monocyte chemoattractant protein-1 induction. J Immunol. 2010 4 15;184(8):4479–87. 10.4049/jimmunol.0901942 20228199 PMC2858914

[36] Kimizuka Y, Kimura S, Saga T, Ishii M, Hasegawa N, Betsuyaku T, et al Roles of interleukin-17 in an experimental Legionella pneumophila pneumonia model. Infect Immun. 2012 3;80(3):1121–7. 10.1128/IAI.05544-11 22144493 PMC3294673

[37] McNamee KE, Alzabin S, Hughes JP, Anand P, Feldmann M, Williams RO, et al IL-17 induces hyperalgesia via TNF-dependent neutrophil infiltration. Pain. 2011 8;152(8):1838–45. 10.1016/j.pain.2011.03.035 21507574

[38] Dumoutier L, Louahed J, Renauld JC. Cloning and characterization of IL-10-related T cell-derived inducible factor (IL-TIF), a novel cytokine structurally related to IL-10 and inducible by IL-9. J Immunol. 2000a;164:1814–1819. 10657629 10.4049/jimmunol.164.4.1814

[39] Felonato M, Pina A, de Araujo EF, Loures FV, Bazan SB, Feriotti C, et al Anti-CD25 treatment depletes Treg cells and decreases disease severity in susceptible and resistant mice infected with Paracoccidioides brasiliensis. VL. PLoS One. 2012;7(11):e51071 10.1371/journal.pone.0051071 23226464 PMC3511355

[40] Clark RA. Skin-resident T cells: the ups and downs of on site immunity. J Invest Dermatol. 2010 2;130(2):362–70. 10.1038/jid.2009.247 19675575 PMC2922675

[41] Matsumoto K, Hashimoto K, Hashiro M, Yoshimasa H, Yoshikawa K.J Modulation of growth and differentiation in normal human keratinocytes by transforming growth factor-beta. Cell Physiol. 1990 10;145(1):95–101. 10.1002/jcp.1041450114 2211846

[42] Silva AA, Sotto MN, Duarte MI, Pagliari C. Regulatory T cells in cutaneous lesions of patients with Paracoccidioidomycosis. Microb Pathog. 2013;65:36–40. 10.1016/j.micpath.2013.09.004 24107311

[43] Ferreira MC, de Oliveira RT, da Silva RM, Blotta MH, Mamoni RL. Involvement of regulatory T cells in the immunosuppression characteristic of patients with paracoccidioidomycosis. Infect Immun. 2010;78(10):4392–401 10.1128/IAI.00487-10 20643858 PMC2950362

[44] Moreira AP, Cavassani KA, Massafera Tristão FS, Campanelli AP, Martinez R, Rossi MA, et al CCR5-dependent regulatory T cell migration mediates fungal survival and severe immunosuppression. J Immunol. 2008 3 1;180(5):3049–56. 18292527 10.4049/jimmunol.180.5.3049

[45] Cavassani KA, Campanelli AP, Moreira AP, Vancim JO, Vitali LH, Mamede RC, et al Systemic and local characterization of regulatory T cells in a chronic fungal infection in humans. J Immunol. 2006 11 1;177(9):5811–8. 17056505 10.4049/jimmunol.177.9.5811

[46] Taylor A, Verhagen J, Blaser K, Akdis M, Akdis CA. Mechanisms of immune suppression by interleukin-10 and transforming growth factor-beta: the role of T regulatory cells. Immunology. 2006 4;117(4):433–42. 16556256 10.1111/j.1365-2567.2006.02321.x PMC1782242

[47] Costa TA, Bazan SB, Feriotti C, Araújo EF, Bassi Ê, Loures FV. In pulmonary paracoccidioidomycosis IL-10 deficiency leads to increased immunity and regressive infection without enhancing tissue pathology. PLoSNegl Trop Dis. 2013 10 24;7(10):e2512 10.1371/journal.pntd.0002512 eCollection 2013. PMC3812093 24205424

[48] Bettelli E, Carrier Y, Gao W, Korn T, Strom TB, Oukka M, Weiner HL, Kuchroo VK. Reciprocal developmental pathways for the generation of pathogenic effector TH17 and regulatory T cells. Nature. 2006 5 11;441(7090):235–8. Epub 2006 Apr 30. 16648838 10.1038/nature04753

[49] Miossec P, Kolls JK. Targeting IL-17 and TH17 cells in chronic inflammation. Nat Rev Drug Discov. 2012;11(10):763–76. 10.1038/nrd3794 23023676

[50] Kennedy J. Mouse IL-17: a cytokine preferentially expressed by alpha beta TCR+CD4+ CD8-T cells. J Interferon Citokine Res. 1996; 16(8):611–617. 10.1089/jir.1996.16.611 8877732

[51] Jin W, Dong C. IL-17 cytokines in immunity and inflammation. Emerg Microbes Infect. 2013 9; 2(9): e60 10.1038/emi.2013.58 26038490 PMC3820987

[52] Kanashiro-Galo L, Pagliari C, Barboza TC, de Brito AC, Xavier MB, de Oliveira CM, et al Th17 and regulatory T cells contribute to the in situ immune response in skin lesions of Jorge Lobo's disease. Med Mycol. 2015 9 1 pii: myv069. 26333354 10.1093/mmy/myv069

[53] Campbell DJ, Koch MA. Treg cells: patrolling a dangerous neighborhood. Nat Med. 2011 8 4;17(8):929–30. 10.1038/nm.2433 21818088

[54] Sakaguchi S, Miyara M, Costantino CM, Hafler DA. FOXP3+ regulatory T cells in the human immune system. Nat Rev Immunol. 2010 7;10(7):490–500. 10.1038/nri2785 Epub 2010 Jun 18. 20559327

[55] Whibley N, Gaffen SL. Brothers in Arms: Th17 and Treg Responses in Candida albicans. Immunity. 2014 4;10(12):e1004456. 10.1371/journal.ppat.1004456 PMC4256420 25474407

[56] De Luca A, Montagnoli C, Zelante T, Bonifazi P, Bozza S, Moretti S, et al Functional yet balanced reactivity to Candida albicans requires TRIF, MyD88, and IDO-dependent inhibition of Rorc. J Immunol. 2007 11 1;179(9):5999–6008. 17947673 10.4049/jimmunol.179.9.5999

[57] Chen X, Oppenheim JJ. Th17 cells and Tregs: unlikely allies. 2014 2 21. [Epub ahead of print]. 10.1189/jlb.1213633 PMC3984971 24563509

[58] Koguchi Y, Kawakami K. Cryptococcal infection and Th1-Th2 cytokine balance. Int Rev Immunol. 2002 Jul-Oct;21(4–5):423–38. 12486822 10.1080/08830180213274

[59] Hube B. Fungal adaptation to the host environment. Curr. Opin. Microbiol. 2009:12,347–349. 10.1016/j.mib.2009.06.009 19577508

[60] Bourgeois C, Kuchler K. Fungal pathogens a sweet and sour treat for toll-like receptors. Front Cell Infect Microbiol.2012;22;2:142 10.3389/fcimb.2012.00142 23189270 PMC3504294

[61] Gordon S, Martinez FO. Alternative activation of macrophages: mechanism and functions. Immunity. 2010 5 28;32(5):593–604. 10.1016/j.immuni.2010.05.007 20510870

[62] Ulges A, Klein M, Reuter S, Gerlitzki B, Hoffmann M, Grebe N, et al Protein kinase CK2 enables regulatory T cells to suppress excessive TH2 responses in vivo. Nat Immunol. 2015 3;16(3):267–75. 10.1038/ni.3083 25599562

[63] Vinh DC. Insights into human antifungal immunity from primary immunodeficiencies. Lancet Infect Dis. 2011 10;11(10):780–92. 10.1016/S1473-3099(11)70217-1 21958581

[64] McCormick LL, Zhang Y, Tootell E, Gilliam AC. Anti-TGF-beta treatment prevents skin and lung fibrosis in murine sclerodermatous graft-versus-host disease: a model for human scleroderma. J Immunol. 1999 11 15;163(10):5693–9. 10553100

[65] Järvinen TA, Ruoslahti E. Targeted Antiscarring Therapy for Tissue Injuries. Adv Wound Care (New Rochelle). 2013 3;2(2):50–54. 24527325 10.1089/wound.2011.0299 PMC3623574

[66] Holmer SM, Evans KS, Asfaw YG, Saini D, Schell WA, Ledford JG, et al Impact of Surfactant Protein D, Interleukin-5, and Eosinophilia on Cryptococcosis. Infect Immun. 2014 2; 82(2): 683–693. 10.1128/IAI.00855-13 24478083 PMC3911392

[67] Piehler D, Stenzel W, Grahnert A, Held J, Richter L, Köhler G, et al Eosinophils Contribute to IL-4 Production and Shape the T-Helper Cytokine Profile and Inflammatory Response in Pulmonary Cryptococcosis. Am J Pathol. 2011 8; 179(2): 733–744. 10.1016/j.ajpath.2011.04.025 21699881 PMC3157286

